# Policy coherence of price controls on food and noncommunicable disease prevention, WHO South-East Asia and Western Pacific regions

**DOI:** 10.2471/BLT.24.291812

**Published:** 2024-11-06

**Authors:** Bella Sträuli, Anne Marie Thow, Erica Reeve

**Affiliations:** a The George Institute for Global Health, Level 18, International Towers 3, 300 Barangaroo Avenue, Sydney, New South Wales 2000, Australia.; b Menzies Centre for Health Policy and Economics, The University of Sydney, Sydney, Australia.; c Institute for Health Transformation, Deakin University, Barwon Heads, Australia.

## Abstract

Noncommunicable diseases are the leading cause of death and disability globally, with suboptimal diet being a significant risk factor. Fiscal policies that promote nutritious foods have been identified as part of a best-practice package of interventions and are a focus for governments in the current context of rising food prices. Price controls are a strategy that governments commonly apply to limit mark-up on prices of specific foods, with the aim of protecting consumers and promoting food security. To date, which specific foods are being placed under price controls is unclear. This paper aimed to provide an overview of the use of food price controls in 10 Member States of the World Health Organization South-East Asia and Western Pacific regions, which have price controls on specific food commodities. The types of foods and beverages under price controls differed considerably. Many of these foods and beverages (for example, sugar-sweetened beverages and instant noodles) were not aligned with global recommendations for healthy diets and the prevention of noncommunicable diseases. Price controls are being implemented by government agencies for finance or commerce, which are generally separate from the agencies overseeing the prevention of noncommunicable diseases. Therefore, an opportunity exists for policy-makers to strengthen policy coherence of price controls on food and the prevention of diet-related noncommunicable diseases.

## Résumé

Les maladies non transmissibles représentent la principale cause de décès et d'incapacité dans le monde, un régime alimentaire inadéquat constituant un facteur de risque important. Les politiques fiscales visant à promouvoir les aliments nutritifs ont été identifiées comme élément constitutif d'un ensemble de bonnes pratiques et intéressent les gouvernements dans le contexte actuel, marqué par la hausse des prix des denrées alimentaires. Exercer un contrôle sur les prix est une stratégie que les gouvernements appliquent fréquemment afin de limiter la marge sur les prix de certaines catégories d'aliments, dans le but de protéger les consommateurs et d'améliorer la sécurité alimentaire. À ce jour, les catégories d'aliments faisant l'objet d'un contrôle des prix ne sont pas clairement définies. L'objectif du présent document est d'offrir un aperçu de l'utilisation de ces contrôles dans 10 États Membres des régions d'Asie du Sud-Est et du Pacifique occidental de l'Organisation mondiale de la Santé, qui appliquent ce type de mécanisme sur des denrées alimentaires spécifiques. Nous avons constaté de grandes différences au niveau des catégories de boissons et aliments concernées. Nombre de ces boissons et aliments (par exemple les boissons sucrées et les nouilles instantanées) ne respectaient pas les recommandations mondiales relatives à une alimentation saine et à la prévention des maladies non transmissibles. Le contrôle des prix est mis en place par des agences gouvernementales dont les compétences relèvent de la finance ou du commerce, qui sont généralement séparées des agences chargées de la prévention des maladies non transmissibles. Les responsables politiques ont donc la possibilité de renforcer la cohérence des politiques de contrôle des prix sur les denrées alimentaires, ainsi que la prévention des maladies non transmissibles liées à l'alimentation.

## Resumen

Las enfermedades no transmisibles son la principal causa de muerte y discapacidad en todo el mundo, y la dieta inadecuada es un factor de riesgo importante. Las políticas fiscales que promueven los alimentos nutritivos se han identificado como parte de un paquete de buenas prácticas de intervención y son un foco de atención para los gobiernos en el contexto actual de aumento de los precios de los alimentos. Los controles de precios son una estrategia que los gobiernos suelen aplicar para limitar los márgenes sobre los precios de determinados alimentos, con el objetivo de proteger a los consumidores y promover la seguridad alimentaria. Hasta la fecha, no está claro qué alimentos específicos están sometidos a control de precios. El objetivo de este documento es ofrecer una visión general del uso de los controles de precios de los alimentos en 10 Estados Miembros de las regiones de la Organización Mundial de la Salud para Asia Sudoriental y el Pacífico Occidental, que tienen controles de precios sobre productos alimenticios específicos. Los tipos de alimentos y bebidas sometidos a controles de precios diferían considerablemente. Muchos de estos alimentos y bebidas (por ejemplo, las bebidas azucaradas y los fideos instantáneos) no se ajustaban a las recomendaciones mundiales sobre dietas saludables y prevención de enfermedades no transmisibles. Los organismos gubernamentales de finanzas o comercio, que suelen ser independientes de los organismos que supervisan la prevención de las enfermedades no transmisibles, son los encargados de aplicar los controles de precios. Por lo tanto, los responsables políticos tienen la oportunidad de reforzar la coherencia política de los controles de precios de los alimentos y la prevención de las enfermedades no transmisibles relacionadas con la alimentación.

## ملخص

تعتبر الأمراض غير المعدية هي السبب الرئيسي للوفاة والإعاقة على مستوى العالم، حيث يُشكل النظام الغذائي دون المستوى الأمثل عاملاً ملموسًا للخطر. وقد تم تحديد السياسات المالية التي تروّج للأطعمة المغذية كجزء من حزمة أفضل الممارسات الخاصة بالتدخلات، وهي محور تركيز الحكومات في السياق الحالي لأسعار المواد الغذائية المرتفعة. ضوابط الأسعار هي استراتيجية تطبقها الحكومات بشكل شائع للحد من زيادة أسعار أغذية معينة، بهدف حماية المستهلكين وتعزيز الأمن الغذائي. وحتى اليوم، لا تزال الأغذية المحددة التي تخضع لضوابط الأسعار غير واضحة. تهدف هذه الورقة إلى تقديم نظرة عامة على استخدام ضوابط أسعار المواد الغذائية في 10 دول أعضاء في منظمة الصحة العالمية، في مناطق جنوب شرق آسيا وغرب المحيط الهادئ، والتي لديها ضوابط للأسعار على سلع غذائية محددة. وقد اختلفت أنواع الأطعمة والمشروبات الخاضعة لضوابط الأسعار بشكل كبير. إن العديد من هذه الأطعمة والمشروبات (على سبيل المثال، المشروبات المحلاة بالسكر، والنودلز سريعة التحضير)، لا تتوافق مع التوصيات العالمية بشأن الأنظمة الغذائية الصحية، والوقاية من الأمراض غير المعدية. يتم تنفيذ ضوابط الأسعار بواسطة الهيئات الحكومية الخاصة بالتمويل أو التجارة، وهي منفصلة بشكل عام عن الهيئات المشرفة على الوقاية من الأمراض غير المعدية. وبالتالي، فإن هناك فرصة مهيأة لواضعي السياسات لتعزيز الالتزام بالسياسات فيما يتصل بضوابط الأسعار على الأغذية، والوقاية من الأمراض غير المعدية المرتبطة بالنظام الغذائي.

## 摘要

非传染性疾病是全球死亡和致残的主要原因，而次优饮食是一个重要的风险因素。以促进营养饮食为目标的财政政策已被纳入一揽子干预措施最佳做法，这些政策也是当前食品价格上涨背景下政府关注的重点。价格管制是各国政府通常用于限制特定食品价格上涨的一种策略，其目的在于保护消费者和促进食品安全。截至目前为止，尚不清楚哪些特定食品受到了价格管制。本文旨在概述世界卫生组织在东南亚和西太平洋地区的 10 个会员国通过实施食品价格管制政策，对特定食品进行价格管制的情况。受价格管制的食品和饮料类型差异很大。其中许多食品和饮料（例如含糖饮料和方便面）不符合全球健康饮食和非传染性疾病预防相关建议。政府金融或商业机构正在实施价格管制政策，而这些机构的职能通常与非传染性疾病预防监管机构是分开的。因此，政策制定者可利用此机会来增强食品价格管制和饮食相关非传染性疾病预防相关政策之间的一致性。

## Резюме

Неинфекционные заболевания являются основной причиной смертности и инвалидности во всем мире, а нерациональное питание является значительным фактором риска. Фискальная политика, направленная на поощрение потребления полезных продуктов питания, была определена как часть комплекса мер, основанных на передовом опыте, и в нынешних условиях роста цен на продовольствие она является одним из приоритетов для правительств. Контроль цен – это стратегия, которую обычно применяют правительства для ограничения наценки на конкретные продукты питания с целью защиты потребителей и обеспечения продовольственной безопасности. Какие именно продукты питания попадают под контроль цен, пока неясно. Цель данного документа – представить обзор использования системы контроля цен на продовольствие в 10 государствах-членах Всемирной организации здравоохранения, входящих в регионы Юго-Восточной Азии и Западной части Тихого океана, в которых действует контроль цен на конкретные продовольственные товары. Виды продуктов питания и напитков, на которые распространяется ценовой контроль, существенно различаются. Многие из этих продуктов питания и напитков (например, подслащенные сахаром напитки и лапша быстрого приготовления) не соответствовали глобальным рекомендациям по здоровому питанию и профилактике неинфекционных заболеваний. Контроль за ценами осуществляется государственными финансовыми или торговыми ведомствами, которые, как правило, не связаны с ведомствами, контролирующими профилактику неинфекционных заболеваний. Таким образом, у ответственных лиц есть возможность усилить согласованность политики контроля цен на продукты питания и профилактики неинфекционных заболеваний, связанных с питанием.

## Introduction

Noncommunicable diseases are the main cause of death and disability worldwide.^1^ Unhealthy diets are a major risk factor for noncommunicable diseases.^2^ Globally, diets have transitioned away from traditional diets comprised of unprocessed foods and towards energy-dense diets that are high in added sugar, sodium and fat.^3^^,^^4^ This transition has contributed to increasing the prevalence of noncommunicable diseases around the world.^5^ In 2017, diet-related risk factors for noncommunicable diseases were responsible for 22% (95% uncertainty interval 21–24%) of all adult deaths globally.^5^ Excessive intake of dietary sodium is the leading dietary risk factor for these diseases due to its association with cardiovascular disease and stroke.^5^ Excessive dietary sodium was responsible for more than half of all diet-related noncommunicable disease deaths (3 million deaths) and two thirds of diet-related disability adjusted life years (70 million) in 2017.^5^ Inadequate intakes of whole grains, fruit, nuts and seeds, vegetables and omega-3 fatty acids are also dietary risk factors for noncommunicable diseases,^5^ with diets low in wholegrains one of the leading diet-related risk factors for death.^5^ Saturated fat, found in fatty meat, dairy foods, and hard fats and oils, contributes to cardiovascular diseases by increasing low-density lipoprotein cholesterol.^6^ Trans-fatty acids, found in baked and fried foods, prepackaged snacks, and meat and milk from ruminant animals, are also associated with high low-density lipoprotein cholesterol, with a strong correlation with all-cause mortality, cardiovascular disease and coronary heart disease.^6^ Lastly, high sugar consumption can contribute to tooth decay and obesity, high blood glucose and high blood pressure,^7^ increasing the risk of diabetes and cardiovascular disease.^8^ Governments are taking concerted action to improve diets as part of initiatives to prevent noncommunicable diseases^9^ and, in many settings, these actions are being implemented within a context of multiple forms of malnutrition and pervasive food insecurity.^4^

Affordability and food prices are important influences on food consumption,^10^^,^^11^ together with economic, behavioural, public health and nutritional factors.^3^^,^^12^ Pricing policies, when used appropriately and especially in the case of healthy foods, can make foods more affordable. Thus, the World Health Organization (WHO) has recommended the use of pricing policies as part of a broader policy package for promoting healthy food environments.^13^ A main challenge for governments is designing and implementing policies that tackle food insecurity, hunger and undernutrition, while also ensuring that these policies do not undermine efforts to prevent other diet-related noncommunicable diseases.^14^ For example, some economic policies to reduce food insecurity, such as subsidies and price controls, have focused on ensuring availability of and access to adequate food (calories) with little consideration of nutritional quality.^15^^,^^16^ In many cases, food policy decisions are being made by multiple government agencies, potentially resulting in inconsistency in policy aims and outcomes.^17^^–^^19^ The implementation of strong policies and programmes that can address both under- and overnutrition in a more coherent and well-coordinated way is a global priority.^20^

## Food price controls

Price controls are a strategy used by governments as a food security measure to protect consumers from the effects of inflation, stabilize prices or reduce pricing inequities, such as those associated with remoteness or the protection of farming incomes.^4^^,^^21^ Price control measures assign maximum or minimum prices for specific food commodities, either in the form of a permissible percentage mark-up or a specific price limit. Price controls can reduce the risk of food shortages in the short-term, but they can distort the situation and artificially keep prices low despite cost pressures faced by the producer or wholesaler (for example, shortages or costs of remote transportation).^4^ The specific food items with price controls vary substantially across countries, but evidence suggests that these items often include foods that could be considered unhealthy.^22^^,^^23^ However, little investigation has been done on the implications of price controls for noncommunicable disease prevention.

Furthermore, price control policies and measures have rarely considered diet-related health objectives and are often under the mandate of finance ministries. Therefore, an opportunity exists for involving the health sector in price control decision-making to promote public health, food security and wider consumer benefits. For the health sector to engage effectively in this decision-making process, health policy-makers, professionals and advocates must understand the existing policy frameworks and priorities, and any collaboration needs to be framed in terms of the benefit to both health and consumers.

In this paper, we examined price control measures in countries in the WHO South-East Asia and the Western Pacific regions in relation to recommendations on the prevention of diet-related noncommunicable diseases. We chose the Western Pacific region because countries in that region have undergone a relatively recent dietary transition from traditional and fresh foods towards energy-dense and nutrient-poor foods. This change has corresponded with increases in the rates of noncommunicable diseases.^24^^,^^25^ The transition has been characterized by increased consumption of processed starch-based foods and foods high in sugar, sodium and saturated fats, including sugar-sweetened beverages, packaged snacks, noodles and processed meat.^5^ The transition has also been coupled with a reduction in the consumption of foods beneficial to health, particularly nuts and seeds, fruit and vegetables, and whole grains.^26^ The prevalence of diet-related noncommunicable disease risk factors, including obesity^27^ and hypertension,^28^ is high in the Western Pacific region and, in 2019, more than 12 million deaths were associated with noncommunicable diseases.^25^ These diseases also have a substantial effect on economies, for example, through direct health-care costs and the indirect effect of premature death and disability on society, health systems and households.^29^ Governments in this region are looking at adopting policies that prevent diet-related diseases, while at the same time considering ways to protect vulnerable groups from food insecurity and malnutrition.^29^ However, the governance of nutrition and noncommunicable diseases is complex because different government sectors have different interests and mandates with respect to food and health.^30^ We also assessed policies from Thailand, a Member State of the South-East Asia Region, because the country has a substantive amount of food price controls in place, and offers an interesting exploration of these policies.

We also considered how strategic engagement by public health policy-makers in the design of fiscal policies could ensure that these policies also contribute to improved health and consumer outcomes. In light of the growing recognition by finance and other ministries of the importance of preventing noncommunicable disease, it is an opportune time for public health policy-makers to support improved coherence in policies related to price control measures.

## Country food price controls

To understand the nature and extent of price controls in place, we used a targeted search strategy to identify all countries with current price control measures in place. We used Google to search government websites and legislative databases of Thailand and all 28 countries of the Western Pacific Region, excluding protectorates operating under external legislative systems. Our initial search was between January 2022 and September 2022, with another in July 2023. We considered any legislation still on government websites and any official announcements via the media (without notice of being revoked) to be active, and looked for complementary material on protected commodities. We used an Excel (Microsoft, Redmond, United States of America) sheet to extract information from each policy on the dates of enactment, objectives, provisions and information on governance and oversight, in addition to other details. We drew on evidence-based recommendations to consider the foods included in price control measures with reference to diet-related risk factors for noncommunicable diseases.

We recorded 14 countries with active price control orders in place and were able to find the evidence of food price control lists in 10 countries: Brunei Darussalam, Fiji, Kiribati, Lao People's Democratic Republic, Malaysia, Philippines, Samoa, Solomon Islands, Thailand and Tuvalu. In many settings, price control policies have been in place for many years, with dates of enactment ranging from 1992 to 2021 (Table 1). Countries had different rationales for having price control orders in place. These reasons included to protect consumers from ongoing and long-term pricing shocks and to stabilize prices in periods of excessive demand, for example, during religious festivals (Table 1). For instance, Brunei Darussalam applies price ceilings during Ramadan for foods that increase in demand at that time,^31^ while Malaysia applies a so-called festival season price control scheme six times throughout the year.^32^ Several countries applied price controls to counter upward pressures faced during situations of higher demand or to protect consumers from profiteering (Table 1). Some countries applied price controls to protect rural communities from experiencing higher prices for essential goods due to the cost associated with supplying goods (particularly perishables) to remote settings (for example, Kiribati).^33^ Three countries applied different price controls for locally produced and imported foods to protect domestic producers: Fiji, Samoa and Thailand (online repository).^34^

**Table 1:** Price controls in the WHO South-East Asia and Western Pacific regions

Country	Name of price control act^a^	Implementing government body	Rationale for the price control
Brunei Darussalam	Price Control (Maximum Prices and Charges) (Amendment) Order, 2021), Constitution of Brunei Darussalam Seasonal Maximum Price (SMP) Directive	Department of Economic Planning and Statistics	Help low-income earners
Fiji	Fijian Competition and Consumer Commission (Price Control) (Food Item Prices) Order 2021	Fiji Commerce Commission; Consumer Council of Fiji; and Ministry of for Commerce, Trade, Tourism and Transport	Ensure fair prices in the market**^b^**
Kiribati	Price Ordinance (Cap 75) Price (Regulation) Order (No.3) of 2021	Ministry of Commerce, Industry and Cooperatives	Reduce cost of living in the Outer Islands and improve affordability of major food items
Lao People's Democratic Republic	Decree on the Administration of Prices of Goods and Services No. 474/PM, 2010	Ministry of Industry and Commerce	Support farmers’ income
Malaysia	Price Control and Anti-Profiteering Act 2011 (as of 1 November 2018) Festive Seasons Price Control Scheme (ongoing)	Ministry of Domestic Trade and Consumer Affairs	Prohibit profiteering
Philippines	The Price Act (Amendment), Republic Act No. 10623, 2012 [an act to amend the original Price Act, No. 7581, 1992]	Price coordinating council, made up of representatives across governmental bodies such as Agriculture, Health, Transportation, Justice, and Trade and Industry	Protect consumers
Samoa	Competition and Consumer Act (2016)	Ministry of Commerce, Industry and Labour; and Competition and Consumer Commission	Protect consumers
Solomon Islands	Law of the Solomon Islands Chapter 64 Price Control (1996 edition)	Ministry of Commerce, Industries, Labour and Immigration	Prevent the commercial sector from taking advantage of consumers
Thailand	Price of Goods and Services Act, B.E. 2541 (1999), 2022 edition	Central Committee on the Price of Goods and Services, Department of Internal Trade, Ministry of Commerce	Protect consumers from exploitative prices
Tuvalu	Tuvalu Price Control Act 2008 Revised Edition Cap. 40.40 Price Control (Amendment) Act 2020	Ministry of Finance	NR

NR: no rationale identified; WHO: World Health Organization.

^a^ Permanent title in the legislation and seasonal price control title where applicable.

^b^ With representatives from: trade and industry; agriculture; health; environment and natural resources; local government; transportation and communications; justice; national economic and development authority; consumers council; producers’ sector; trading sector; manufacturers' sector

The price control lists in these countries included eight food categories common in food-based dietary guidelines.^32^ These categories were: (i) starchy staples; (ii) fruit and vegetables; (iii) protein foods; (iv) dairy; (v) oils; (vi) condiments, coffee, tea and water; (vii) discretionary foods; and (viii) other consumable commodities, including formula milk for babies, baby food and water (Table 2). The categories of food that were most commonly protected were starchy staples (nine countries), discretionary foods (nine countries), protein foods (eight countries), dairy products (eight countries) and oils (six countries). The specific foods that countries most often placed price protections on were rice (nine countries), sugar (eight countries), flour and processed milk (eight countries), and oil (six countries). Vegetables, poultry, fresh meat, and canned, corned and processed meat were each protected in five countries.

**Table 2:** Price-protected food groups and food, South-East Asia and Western Pacific regions, 2022

Food group^a^	Countries protecting food group (*n* = 10), no.	Food and beverage elements in the food group	Countries protecting this food type, no.
Starchy staples	9	Rice	9
		Flour, wheat	7
		Starchy vegetables (e.g. potatoes and root crops)	5
		Bread	1
		Cereal	1
Fruit and vegetables	5	Vegetables (e.g. onions, garlic and chillies)	5
		Fruit (e.g. coconut, mangosteen, durian and logan)	3
Protein	8	Poultry (e.g. chicken and goose)	5
		Fresh meat (e.g. pork, beef and buffalo)	5
		Processed meat (e.g. luncheon meat, canned and corned meat)	5
		Frozen goods (e.g. turkey tail, mutton flaps, pork trotter, chicken leg, turkey wings and turkey tail)	1
		Fresh fish (and fish products)	2
		Canned fish (and other marine products)	4
		Eggs	3
		Legumes	1
Dairy	8	Processed milk (e.g. evaporated, powdered and condensed)	8
		Milk (fresh)	4
		Milk (creamer – a processed concentrated powdered milk)	1
		Other dairy products (unspecified)	1
Oils	6	Oils (e.g. canola, corn, palm, vegetable, sunflower and soya bean)	6
Condiments, coffee and tea	3	Cooking sauces (e.g. oyster, chilli, ketchup, soy and fish)	3
		Coffee and tea	3
		Vinegar	1
		Curry powder	1
Discretionary foods and beverages	9	Sugar	8
		Salt	4
		Butter	3
		Margarine	2
		Ghee	1
		Instant noodles	3
		Sugar-sweetened beverages (e.g. coffee 3 in1,^b^ tea 3 in1 and fruit juices with high amounts of added sugar)	3
		Milk (condensed)	3
		Biscuits	2
Other	4	Formula milk for babies and baby food	3
		Water	1

WHO: World Health Organization.

^a^ Food categories were determined from a global review of food based dietary guidelines.^32^

^b^ 3 in1 drinks are considered sugar-sweetened beverages due to the addition of sugar and powdered milk or creamer, as well as additional substances to improve shelf life in some of these products.

In the 10 countries we examined, the control of food pricing was under government agencies with an economic mandate. These agencies included finance, economics and/or commerce (seven countries), internal trade and consumer affairs (two countries), and other agencies implementing price controls, for example, the price coordinating council in one country (Table 1). We could not find any evidence of collaboration between finance or commerce and nutrition and health experts in the development of price control measures.

We identified two types of price controls: (i) a mark-up control, where an implementing body assigns a maximum percentage mark-up (profit) to the given product; and (ii) a set price control, where an implementing body assigns a specific maximum price. 

## Coherence with disease prevention

We identified several cases where core foods normally recommended in food-based dietary guidelines were protected with price controls.^35^ For instance, WHO recommends that countries increase the availability, affordability and consumption of a range of fruits and vegetables as a part of efforts to support the prevention of diet-related noncommunicable diseases.^13^ Five countries in our review were applying price controls to vegetables, three countries were applying them to fruit and one country was applying them to legumes (Table 2). However, the vegetables with price controls tended to be starchy varieties such as root crops (for example, cassava, taro and unspecified root vegetables) and potatoes (Table 2). Additionally, milk, other dairy foods, fresh unprocessed meat, poultry and fish, and eggs are considered core foods within food-based dietary guidelines because of their important contribution to dietary intake of protein and micronutrients (in particular, iron).^35^ Fresh meat (for example, beef, lamb and pork) and poultry products (for example, chicken and goose) had price controls in five countries, while eggs had price controls in three countries and fresh fish had price controls in two countries. Canned fish had price controls in four countries. Although these products are generally considered minimally processed, the sodium content of the available brands may need to be assessed for compliance with the recommended upper limits. Additionally, perhaps price controls could incentivize a switch to brands with lower sodium content.

We also identified several potential inconsistencies between global dietary guidance and the application of price controls. For instance, WHO recommends reducing intake of processed meats due to their association with a range of cancers,^36^ and processed meats are often high in salt and/or saturated fat.^37^ Despite this recommendation, processed meat products (for example, luncheon meat and corned beef) had price controls in five countries. Additionally, salt reduction is considered one of the best-buys for noncommunicable disease prevention,^13^ and WHO recommends that countries aim to reduce population dietary intake of salt and sodium by 30%.^38^ However, table salt had price controls in four countries as did several foods known to contain high levels of sodium, including soy sauce, fish sauce, instant noodles and instant packaged food.^39^ Lastly, WHO’s guideline on sugar intake for adults and children makes a strong recommendation to limit dietary intake of free sugars to less than 10% (ideally 5%) of total energy intake.^38^ However, eight countries had price controls on sugar or foods high in sugar such as sugar-sweetened beverages (three countries), condensed milk (three countries) and biscuits (two countries) (Table 2).

WHO also recommends reducing population dietary intake of saturated fat to < 10% of total energy intake and restricting consumption of trans-fatty acids.^38^ Fats and oils, which are necessary for cooking and provide an important source of energy in some settings,^40^ had price controls in six countries. While protecting the price of healthier oils (for example, oils low in saturated fat or non-hydrogenated oils) could be a strategy for promoting the consumption of healthier oils, some countries specified palm oil or a more generic vegetable oil,^2^ or edible oils of all types,^2^ which in many settings includes partially hydrogenated vegetable oils that are high in trans-fat.^40^ Hydrogenated oils are generally more affordable than healthier alternatives^41^ and the protection of hydrogenated oils in the lower- and middle-income countries studied is likely evidence that they are the main oils being used for affordability reasons.

In many of the countries, we found that processed versions of food had price controls even though a healthier alternative existed. For example, instant noodles, although disproportionately high in sodium, energy and saturated fat, had price controls in three countries. While instant noodles are considered a staple in many countries in the Western Pacific region,^42^ their consumption displaces other staple foods with similar energy but higher levels of fibre and micronutrients (for example root vegetables and wholegrains).

Three countries (Brunei Darussalam, Fiji and Kiribati) were applying price controls to breastmilk substitutes or baby food. The International Code of Marketing of Breast Milk Substitutes states that health systems should not promote low-cost or discount options for breastmilk alternatives.^43^ Although such price controls do not conflict with this recommendation, they convey a perception that infant formula is an essential commodity.

## Need for policy coherence 

The protection of foods high in sugar, salt and saturated fat with price controls contradicts WHO recommendations to promote healthy diets and is an example of incoherence between health and economic policy. This lack of coherence creates several challenges for government. First, the protection of foods that are not recommended for healthy diets can lead to regulatory confusion. For example, Fiji and Samoa had policy initiatives to reduce population salt consumption while at the same time-limiting the price of salt.^44^ Second, price controls are used to protect consumers from paying inflated prices for essential commodities. Thus, price controls for less healthy foods and beverages may signal to consumers that these unhealthy foods are considered essential. Third, price controls for unhealthy foods may undermine the effectiveness of policies where governments have adopted fiscal policies (for example, taxes) aiming at reducing their consumption.^38^ For example, Fiji, Kiribati, Samoa, the Solomon Islands and Tuvalu have taxes on sugar-sweetened beverages but have also applied price controls to sugar and/or sugar-sweetened beverages.^45^ We did not identify any intentional or public regulatory policies to address this incoherence, however governments may already be considering this issue as part of internal discussions.

## Way forward

Several opportunities exist for public health policy-makers to enhance the effectiveness of noncommunicable disease prevention measures and create more consistent messages for consumers by identifying and using price control measures.

First, in the context of a noncommunicable disease epidemic, a review of price control policies is urgently needed to assess their coherence with public health. In addition, other public policies that no longer support broader government objectives for promoting nutrition and development need to be evaluated, in line with global objectives for sustainable development, in particular, sustainable development goal 17, target 17.14: enhance policy coherence for sustainable development. The broader transformation agenda for food systems emphasizes the need to strengthen and foster institutional mechanisms that can assess the effect of existing government policy instruments on the goals of food systems.^46^ The establishment of interministerial governance mechanisms associated with the United Nations Food Systems Summit, including the national food systems dialogues, provides a new opportunity for health policy-makers to advocate for assessment of government policy instruments for coherence with priorities to tackle all forms of malnutrition, including diet-related noncommunicable disease risks. While health policy-makers are generally closely involved in nutrition policy design and governance, their authority to engage in the decisions of other sectors that affect food consumption and nutrition and the opportunities for them to do so need to be strengthened.^26^ The engagement of health stakeholders in decisions on agricultural and food pricing measures could help to identify specific instances where price controls may be undermining health policy objectives. By engaging with this broader agenda, public health policy expertise can contribute to more effective use of pricing measures in all areas to advance health, and livelihood-related and environmental policy objectives.

Second, an opportunity also exists to maintain the use of price controls in ways that contribute to broader health and social policy objectives. This approach fits with global priorities to improve pricing and subsidy measures for agriculture and food to more holistically promote environmental sustainability, social and economic livelihoods, and nutritious food supply.^4^^,^^47^ Price controls can also be used as an equity measure to protect consumers (for example, Brunei Darussalam price controls protect low-income earners, especially in festive seasons when prices are anticipated to increase)^48^ and farmers (for example, Lao People's Democratic Republic has adopted price controls to protect farmers’ incomes).^49^ By more strongly considering nutrition as a determinant in the selection of foods for price controls, such controls could be a policy tool to encourage consumers to make healthier food choices.^4^ For instance, in the case of fats and oils, price controls could be an incentive for consumers to switch to healthier non-hydrogenated alternatives, such as canola oil. Investing in the evaluation of these measures would provide further information on the effectiveness of this policy approach.

In conclusion, while price controls could be used in ways that promote the sustainable development agenda, protecting the price of foods high in sodium, sugar or fat is inconsistent with dietary recommendations and therefore conflicts with goals to promote healthy population diets.^46^ Greater collaboration is needed between health and finance arms of governments in the design and application of economic policies that aim to promote health.^30^ Health policy-makers can capitalize on the momentum of the United Nations Food Systems Summit as a platform for engaging in policies related to food systems that might promote nutrition and food security, in addition to other outcomes. Further research is needed to improve our understanding of how pricing policies affect consumption and the complementary policy measures that can be used to ensure healthier foods are affordable for the general population.
